# Brain Share, Not Brain Drain

**DOI:** 10.1200/GO.20.00069

**Published:** 2020-03-26

**Authors:** Vinay Mathew Thomas, Aju Mathew

**Affiliations:** Vinay Mathew Thomas, MBBS, Department of Internal Medicine, University of Connecticut Health Center, Farmington, CT; and Aju Mathew, MD, MPhil, University of Kentucky Markey Cancer Center, Lexington, KY and Malankara Orthodox Syrian Church Medical College Kolenchery, Kerala, India

The article by El Saghir et al^1^ describes how governments of different countries have attempted to decrease the emigration of their physicians. The authors discuss how India has attempted to do the same by issuing a No Obligation to Return to India (NORI) certificate. The NORI certificate applies only to a specific type of visa, J-1, the exchange visitor program visa, and cannot be applied to a large percentage of Indian physicians who are on other visas. It is not intended to reduce the migration of physicians; it is just a response to a mandatory requirement from the US government to waive off the original agreement made by professionals that they will return to their home countries for at least 2 years. NORI certification allows for emigration. We think it is up to the individual to choose his or her vocation and where he or she wishes to practice it, and we therefore support the Indian government’s NORI certification.

The concept of brain drain is interesting; however, we would like to think of it differently. We would like to put forth the idea of brain share, rather than brain drain. Just because a few doctors leave a nation does not mean that the brain power in that country is drained. Furthermore, we do not think that a nation receiving doctors from another country had been devoid of brain power. It is an unproven assumption that the best brains migrate; that is a misnomer. In fact, there are numerous factors that lead to the decision to migrate. There is certainly a differential balance when it comes to manpower. There are solutions that can enrich the home and host countries. For instance, India has opened up its medical education sector to private investment, and the number of medical school training positions has increased; however, a proportionate increase in the number of postgraduate training opportunities has lagged behind. There is rightfully a greater focus on rectifying this imbalance. Conversely, a host nation can invest in medical education in the home country via need-based scholarship programs. We have rarely seen such a program from a host nation.

Even with the migration of physicians from their home countries, there is a profusion of proficient physicians who stay behind who take up the mantle of striving toward excellence in health care. Even so, alumni who emigrate can enrich their home countries through various activities. For instance, the Association of Kerala Medical Graduates, an alumni organization of medical professionals from the state of Kerala in India, is joining hands with the Association of Medical and Pediatric Oncologists of Kerala to organize the Kerala Cancer Conclave in September 2020. Our aim is to reduce the cancer burden in this the region with the highest incidence of cancer in India. The research and philanthropic support that can be provided by alumni will help fill some gaps in their home nation and translate to meaningful improvement in the lives of its people.

Physical and imaginary boundaries are disappearing as a result of the great information superhighways. Greater exchange of intellect results in profound benefits for the global populace. Doctors may relocate from a home to a host country, but they are still in the macrocosm of the international physician community. Therefore, we propose that there is no draining of talent, there is just a sharing of brains.

## References

[1] El Saghir NS Anderson BO Gralow J et al Impact of merit-based immigration policies on brain drain from low- and middle-income countries JCO Glob Oncol 6 185 189 2020 32023124 10.1200/JGO.19.00266 PMC7051246

